# Enhanced Immune Responses by Virus-Mimetic Polymeric Nanostructures Against Infectious Diseases

**DOI:** 10.3389/fimmu.2021.804416

**Published:** 2022-01-19

**Authors:** Xinpei Li, Shengqiu Liu, Panchao Yin, Kun Chen

**Affiliations:** ^1^ South China Advanced Institute for Soft Matter Science and Technology, School of Molecular Science and Engineering, South China University of Technology, Guangzhou, China; ^2^ State Key Laboratory of Luminescent Materials and Devices & Guangdong Provincial Key Laboratory of Functional and Intelligent Hybrid Materials and Devices, South China University of Technology, Guangzhou, China

**Keywords:** subunit vaccines, virus-mimetic polymeric nanostructures, multivalent epitope, molecular adjuvants, enhanced immune responses

## Abstract

Intermittent outbreaks of global pandemic disease have spurred new sensors and medicines development for the prevention of disease spread. This perspective specifically covers recent advances, challenges, and future directions in virus-mimetic polymeric nanostructures and their application in biological medicines with a special emphasis on subunit vaccine development. With tailorable compositions and properties, polymers facilitate the ingenious design of various polymeric nanostructures. As one type of polymeric nanostructures, virus-mimetic polymeric nanostructures have been developed as an attractive platform for enhanced immune responses, since they combine the merits of polymer nanocores with the biomimetic characteristic of virus which displays multivalent epitopes on their surfaces. This perspective also provides an applicative approach to rationally design virus-mimetic polymeric platforms based on nanostructures that are self-assembled by using polymers as templates and the antigens and metal oxide clusters loaded on their surface to mimic viruses in size and surface antigenicity. Sub-200 nm virus-mimetic polymeric nanostructures are in a relatively lower level of endotoxins and can promote the antigens to elicit potent humoral and cellular immune responses against pathogenic bacteria. The promising development of virus-mimetic polymeric nanostructures will continue to protect human health from common pathogens and emerging infectious threats.

## Introduction

Vaccination activates immune responses against infectious disease by prevention of infection, reduction in disease severity or the rate of hospitalization, which has been demonstrated in improving global human health (1, 2). In general, vaccines are classified as live, non-live or other recent developed platforms, such as virus-like particles (VLPs), nucleic acid-based (RNA and DNA) vaccines, and subunit vaccines. Live and live attenuated vaccines still raise safety concerns due to the potential reversion to their pathogenic forms that are capable of replicating in an uncontrolled manner (3). These issues are particularly aggravated in immunocompromised individuals, which restricts the wide application of live vaccines. Non-live vaccines are composed of antigenic proteins or polysaccharides from the organism, recombinant proteins, or the killed whole organisms, and can provide comparable and beneficial immunity effects in most cases. For instance, inactivated whole virus vaccines (InWVV) induce humoral immunity and generate high-titer specific IgG antibodies, except that cellular immune response induced by InWVV is usually too low to provide effective and long-lasting protection. Excessive stresses have been imposed on vaccinated animals because of the high immunizing doses and potential severe side effects of InWVV (4–8). These disadvantages dampen the enthusiasm of scientists for the design of InWVV.

One class of nucleic acid vaccines, mRNA vaccine, the sequence of which can code a specific protein, represents a promising alternative to conventional vaccine by modulating the post-translational modifications and inducing transient protein expression. Because of the beneficial features above-mentioned, mRNA has been developed rapidly and become mature in the fields of nucleic acid vaccines (9). Various materials, including lipids, lipid-like materials, polymers, and hybrid systems, have been applied for mRNA delivery (10, 11). BNT162b2 and mRNA-1273 vaccines are lipid nanoparticle-formulated both of which have shown high efficacy at preventing the coronavirus disease 2019 (COVID-2019) (12, 13). Polymeric nanoparticles deliver antigen-encoding replicon mRNA into mice, so that the mounted cellular and antibody responses protect mice from H1N1 influenza and Ebola virus as well as *Toxoplasma gondii* parasite (14). Delivered by polymeric nanoparticles, mRNA molecules specifically reach target cells and sufficiently produce interest proteins. Nonetheless, high production costs and safety problems are still challenging for mRNA vaccine (15). Thus, safe and effective mRNA delivery materials are in urgent need of vaccine development.

Subunit vaccines pose no risk to the immunocompromised individuals since they have lost the potential to replicate in an uncontrolled manner (16). However, a trade-off lies between strong immunogenicity and sufficient safety. To improve the ability to induce an immune response, subunit vaccines are often combined with an adjuvant which can enhance the immune response against the antigen by providing danger signals to the innate immune system. Emergence of new adjuvanted vaccines suggests that the provision of additional signals to the immune system by certain adjuvants can overcome the decline in immune function (17). Among them, polymeric nanostructures as vaccine platforms have attracted special attention due to their high tailorability. This perspective will focus on the virus-mimetic polymeric nanostructures (VMPNs) as vaccine platforms, exemplify virus-mimetic polymeric platforms for subunit vaccines, and discuss the challenges faced by virus-mimetic polymeric vaccines. Moreover, a virus-mimetic nanostructure composed of polymers and molecular adjuvants is presented in the end of this perspective, aiming to demonstrate the convenience and flexibility of co-assembly strategy in designing polymeric nanostructure-based subunit vaccines.

## Polymeric Delivery Vehicles

**Figure 1:**
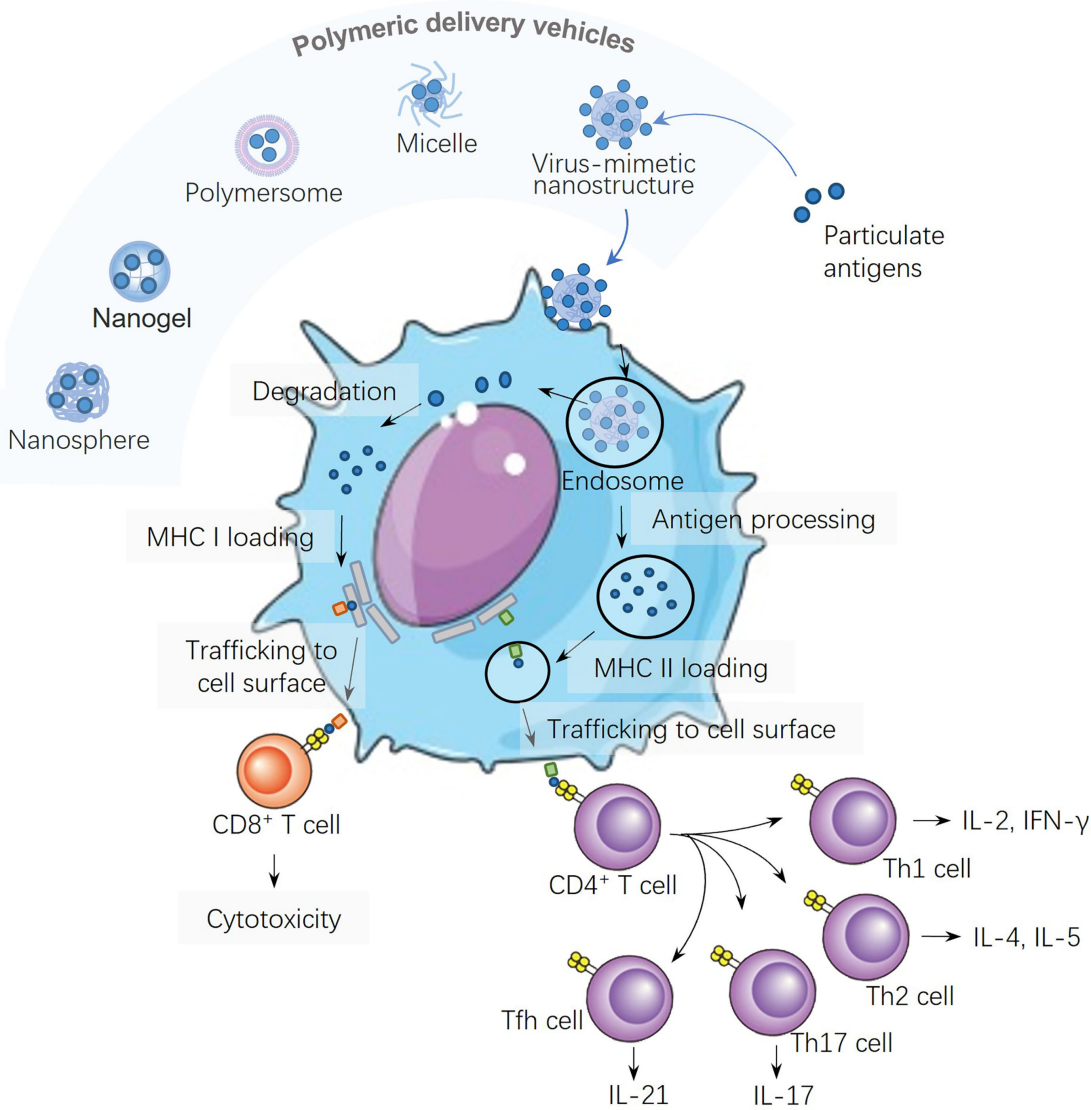
Schematic illustration of different polymeric structures for vaccine development and the antigen processing and presentation of virus-mimetic polymeric nanostructures in a dendritic cell.

Owing to rich chemistry (tailorability), biocompatibility and biodegradability, polymer-based nanostructures have been developed against various emerging diseases. Nanostructures based on polymers are promising vehicles (18) to transport medicines, contrast agents, and gene vectors. For example, a pH-responsive polymer nanoparticle was designed to respond to acidic lysosomes by increases in diameters from 200 to 500 nm when the pH was dropped from 7.4 to 4.9 to disrupt the membranes of acidic lysosomes for cytosolic drug delivery (19). Most importantly, polymeric nanostructures can be modularized as adjuvants to adopt immunological cues by mimicking biophysical and biochemical characteristics of pathogens to elicit robust and protective immune responses upon vaccination. Polymer-based delivery vehicles include solids, polymeric nanospheres, nanogels, polymersomes (20), micelles, and virus-mimetic nanostructures (**Figure 1**), where various antigens are encapsulated into the core or displayed onto the surface. The polymeric delivery strategy can load/assemble not only antigens but also molecular adjuvants into the particulates.

## Virus-Mimetic Polymeric Nanostructures

Polymeric delivery vehicles provide strategies for engineering the antigenic components to particular immune cells and lymphatic tissues. The physical and biochemical features of polymeric delivery vehicles, including size and shape of the polymeric delivery vehicles, the number and physical position of the antigens, can be engineered to modulate their cellular uptake and tissue distribution (21, 22). VLPs are nanostructures constructed of viral proteins that mimic the authentic virus but lack the viral genome. Analogously, polymeric nanostructures decorated with spikes, such as VMPNs, have unique impacts on immune activation. Spatial repetition on surface of VMPNs is an intrinsic feature to virus, meanwhile, immune system of recipients has evolved to recognize the feature and respond to it with high sensitivity. Therefore, virus-mimetic nanostructures are engineered to advance the vaccine strategies against infectious diseases.

In nanomedicine and nanopharmacology, virus-mimetic nanoparticles are designed by mimicking viral assembly for advanced disease diagnosis and therapy (23, 24). Virus-mimetic polymeric micelles that carried dual-functional moieties on their surfaces for cell-specific recognition and enhanced cell penetration, were developed for cancer therapy (Xiong and Lavasanifar, 2011). Decorating nanoparticles with protein antigens or recombinant antigens as a simple approach was frequently proposed. Generally, VMPNs for vaccine delivery are facilely obtained based on the self-assembly of the polymers with protein antigens and adjuvants in solutions. The physiochemical properties of polymers can be fine-tuned by advanced polymer synthesis and processing technologies. Engineered polymers are programmed to organize into specific nanoarchitectures that are responsive upon exposure to various external stimuli for efficient vaccine delivery (25). VMPNs have been employed to develop nanodelivery strategies for enhanced cancer immunotherapy (26, 27) based on the property of polymeric nanostructures that can direct immunomodulators to tumors and lymphoid organs. VMPNs also can be engineered with multivalent interaction sites, improving their engagement with the immune system.

## Antigen Processing and Presentation in Dendritic Cells

Antigens carried in polymeric particles can be digested by dendritic cells (DCs), and the resultant antigen peptide fragments are presented on the surfaces of DCs against the backdrop of major histocompatibility complexes (MHCs). Two pathways for antigen presentation are shown in **Figure 1**, both of which have significant roles in inducing humoral and cellular immunities: (i) the class II MHC (MHC II) antigen presentation pathway for CD4^+^ T cell activation, and (ii) the class I (MHC I) antigen cross-presentation pathway for CD8^+^ T cell activation. Nanoparticles can be used to modulate the lymph node follicles capture and antigen retention to induce germinal centers and long-lived humoral immunity. Large nanoparticles are more like to be opsonized by complements more than small ones, which lead to enhanced nanoparticle retention, antigen presentation on follicular DCs, and more robust germinal center reactions (28). Therefore, the density and spatial distribution of surface antigens distinctly affect their ability in inducing T helper cell responses, since multivalent interactions are required for fully eliciting downstream signaling pathways (29).

It has been demonstrated that polymeric nanovaccines based on protein-delivering dendrimers show promise for effective antigen cross-presentation and cancer immunotherapy (30). Appropriate packaging of antigens is critical to increase their presentation to DCs, and to prevent the systemic toxicity of the whole vaccines. Surface topography, like spiky nanostructures on viruses, is capable of activating innate immunity during interaction and phagocytosis by DCs. A surface modified polymeric structure consisting of poly(lactic-co-glycolic acid) (PLGA) is able to deliver tumor-specific proteins to antigen-presenting cells (APCs), resulting in an expansion of CD8^+^ cytotoxic T cells and the improved immunotherapeutic effect (31).

## Virus-Mimetic Polymeric Nanostructures in Subunit Vaccines

A subunit vaccine consists of certain antigenic components including antigens based on carbohydrates, lipids, peptides and proteins, or bacterial lysates. Immune response by subunit vaccine differs due to the different antigens carried. Protein antigens usually give rise to T-cell dependent adaptive immune responses, while polysaccharide antigens generate T-cell independent responses. A subunit vaccine with the SARS-CoV-2 spike protein receptor-binding domain stimulated robust and durable neutralizing-antibody response against SARS-CoV-2 in rhesus macaques (32). Fragments of protein shells mimicking the coronavirus’ outer coat also were developed into subunit vaccines, which protected monkeys against the coronavirus infection but haven’t been tested in people (33). No risk is a distinct advantage of subunit vaccines; however, they are less immunogenic than live attenuated vaccines which may not confer protection for the individuals with B cell or combined immunodeficiency. Subunit vaccines with multivalent epitopes have been developed to deal with the weak immunogenic issue. Haemagglutinin inserted at the interface of the adjacent subunit spontaneously assembled and generated eight trimeric viral spikes on its surface. Immunization with this nanoparticle vaccine produced haemagglutinin inhibition antibody titers tenfold more than those from the licensed inactivated vaccine (34). Various particulate carriers have been developed for subunit vaccines, and understanding the fluid dynamics of these carriers is the key to improving the bio-distribution and antigen presentation (15).

To enhance subunit vaccine immune responses, a variety of approaches, including the presentation of epitopes in multimeric format (e.g., above-listed nanoparticle vaccine, and VLPs) or the use of immunostimulatory adjuvants, have been designed and utilized in preventing infectious diseases. Virus-mimetic subunit vaccine composes of one or more antigenic proteins that mimic the shape and size of the native virions by co-assembling with adjuvant materials. Since virus-mimetic subunit vaccine is safer than infectious attenuated and inactivated vaccines with the ability to efficiently elicit humoral and cellular immune responses (35, 36), it has been one of the most promising vaccine candidates. Especially, virus-mimetic polymeric nanostructure loaded with various vaccine components in a nano-size range can mimic viral or bacterial not only in size but also in topology, facilitating their co-delivery and the pathogen recognition by APCs. Polymers from the nanostructure can be acted as direct delivery vehicle or delivery vehicle and adjuvants together, or loading with other adjuvants as vaccines. Polymer as the direct delivery vehicle in vaccine platform has been reported as follows: a polymer-templated protein nanoball (PTPNB) with controlled orientation of hemagglutinin 1 (H1) on its surface was designed at the similar size with viruses without the addition of adjuvant. H1-PTPNB efficiently promoted H1-specific immune activation and cross-protective activities, due to the exposure of H1 head group on the surface and a similar size to that of influenza virus (37).

The protein-coated polymeric structure displays protein antigens with controlled orientation and repetitive structures mimicking the surface features of virus or a bacterium *via* the self-assembly of the polymers either *in viro* in bulk solutions or *in viro* in engineered bacteria. Poly(ϵ-caprolactone) (PCL) was grafted with pyridine, which provided a docking site for linking PCL with the dengue virus serotype-2 envelope protein (DV2EP) or *Plasmodium falciparum* malaria circumsporozoite protein (CSP) antigens through hydrogen bonding upon nanoparticle formation (38, 39). For DV2EP and CSP mice groups, the trials demonstrated significant increases of antigen-specific IgG1 and IgG2a titers compared with the antigens alone. As an emerging material for particulate vaccines, the bacterial biopolymer poly(3-hydroxybutyric acid) (PHB) enables rapid design and cost-effective manufacture at scale. To date, protein-coated PHB particles have been developed as a vaccine platform for the delivery of infectious-disease associated antigens originated from viruses (e.g., hepatitis C virus) and bacteria (e.g., *Streptococcus pneumoniae*, *Mycobacterium tuberculosis*, and *Neisseria meningtidis*). Mice immunized with antigen-coated PHB particles demonstrated robust humoral- and cell-mediated immune responses. In an attempt to develop vaccines against tuberculosis, multi-antigenic PHB composed of three *Mycobacterium tuberculosis* (TB) antigens (H28) and adjuvanted with dioctadecyl ammonium bromide (DDA) micelles was subcutaneously injected into mice. This formular induced highly antigen-specific IgG1 and IgG2c titers with strong cytokine profiles (40). The multi-antigenic features and large scale manufacture make PHB particle vaccine strategy suite to respond to pandemic causing SARS-CoV-2 (41). A polyamidoamine dendrimer modified with guanidinobenzoic acid (DGBA) was reported to serve as an effective protein antigen carrier. DGBA allows efficient surface adsorption of the antigen proteins to form core-shell nanoparticles (30) that show enhanced intracellular delivery and effective endosomal/lysosomal escape, thereby leading to effective antigen cross-presentation by DCs. Apparently, this virus-mimetic hierarchical structure synergistically combines the merits of polymer cores (physical stability, antigen-encapsulation space, payload for molecular adjuvants and controlled release properties) with the biomimetic characteristics of bacterial or viral surfaces, to enhance antigen-specific immune responses. The rational design of virus-mimetic polymeric nanostructure platforms for subunit vaccines meets the need to achieve control over humoral and cell-mediated immune responses.

## Challenges and Perspective

In preclinical studies, polymeric nanostructures against many diseases show promising futures, but only a few have met the standards of the clinical applications. It is challenging for polymeric delivery vehicles to achieve clinical application and ultimately reach the market because of their weak polymeric nanostructures. First, the complex layers of polymeric nanostructures pose huge challenges, including scaling up production, setting wallet-friendly prices, and maintaining batch-to-batch reproducibility. To reach clinical trials, poly(ethylene imine) (PEI) polyplexes for HIV are synthesized by the self-assembly between PEI with cationic charges and DNA with anionic charges, which exhibits scale-up and process simplicity (42). Thus, a simple fabrication process is the preferred method. Second, several polymeric delivery vehicles composed of temperature-sensitive units (e.g., recombinant protein antigens) keep their stability *via* cold chains, which brings problems to be solved in global distribution, storage, and administration. To reduce dependence on cold chains, single-dose polymeric implant *via* melt-processing (43), microneedle-based patches (44), and thin film-based vaccines (45) are developed. Third, safety significantly does matter in this issue. For several polymers, the biodegradation products may stir up cyto-toxicity and unexpected nonspecific immune responses. Last but not least, long-lasting protective immunity needs to be established for inducing strong and specific immune responses. Polymeric delivery vehicles served as rapid response platforms have been fast developing, aiming to reach the market and combat the emerging pandemic threats.

## Discussion

**Figure 2:**
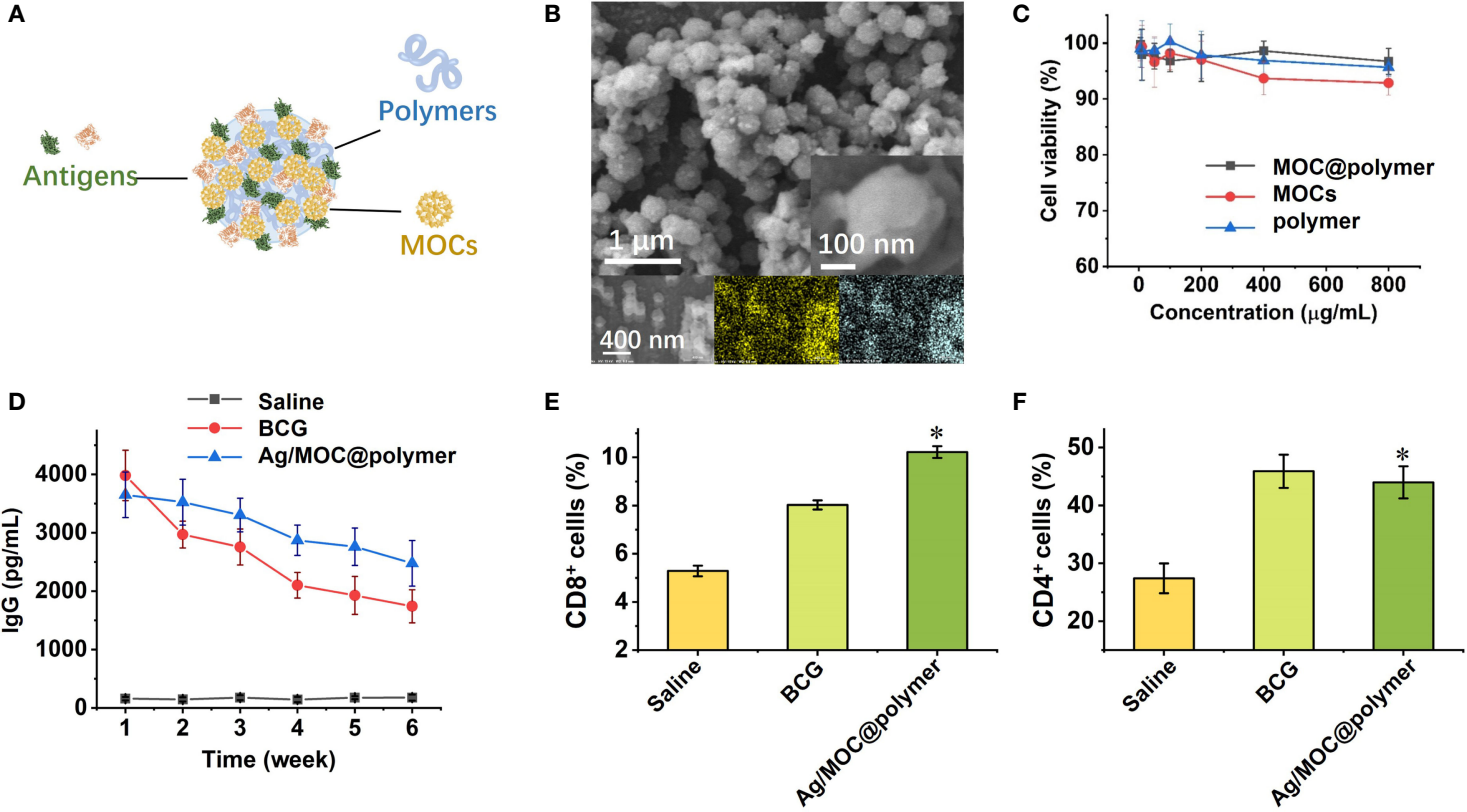
**(A)** Schematic illustration of self-assembly of virus-mimetic polymeric structures (Ag/MOC@polymer supra-molecular particle assemblies; Ag abbreviated from antigens) for the construction of subunit vaccine (50). **(B)** SEM image of virus-mimetic polymeric structures. Inset: magnified SEM image of one virus-mimetic polymeric structure. **(C)** Cytotoxicity of virus-mimetic polymeric structures on murine bone marrow derived dendritic cells (BMDC) assessed using the colorimetric cell counting kit-8 (CCK-8). **(D)** Serum TB-specific IgG titres over time after inoculation of virus-mimetic polymeric structures. Count rates of CD8^+^ **(E)** and CD4^+^ cells **(F)** in Balb/c mice spleen by flow cytometry one week after inoculation. Each data shows mean ± s.e.m. from a representative experiment (n = 6 for each group) out of three independent experiments. *p < 0.05, are analyzed by one-way ANOVA. * Indicates statistically significant differences between tested groups and saline groups.

There is a lack of clinically approved adjuvants that can elicit antigen-specific effector and long-lived CD4^+^ and CD8^+^ T cells (46). To design vaccines that recapitulate the efficacy of nanostructures and metal oxide clusters (MOCs), biocompatible polymers are used as templates to load ultra-small MOCs and antigens separated from pathogens to co-assemble the nanostructures. MOC is rich in hydrogen bonding donor sites (47), and it shares the common structural feature with most biomacromolecules which can bridge MOCs with polymers *via* multiple hydrogen bonds onto the co-assemblies (48, 49). Thus, the co-assembly profile provides a universal method (**Figure 2A**) to construct colloidal nanostructures serving as complex nano-vaccines (50), which carry two moieties (MOCs as the molecular adjuvants and proteins extracted from pathogens as the subunit antigens).

Due to their high surface areas, the synthesized virus-mimetic co-assemblies exhibit high antigen loading and entrapment efficiency. Scanning electron microscopy (SEM) studies confirm that the virus-mimetic co-assemblies possess uniform size distribution with distinct roughness (**Figure 2B**), since the antigens and MOCs were closely packed on the surface of virus-mimetic co-assemblies. Cytotoxicity tests (**Figure 2C**) verify that MOCs and the used polymers are safe as vaccine adjuvants. The antigens presented on the surfaces provide multivalent epitopes for enhancing local DCs uptake and subsequent antigen presentation, followed by the activation and differentiation of naive T lymphocytes, CD4^+^ Th cells and CD8^+^ cells. Humoral immunity inducing antibody-mediated immune responses is mainly regulated by B lymphocytes. While T lymphocytes regulate the cellular immunity and induce cell-mediated immune responses. Mice inoculated with the virus-mimetic co-assemblies have almost equal titers of antibodies compared with BCG group over time (**Figure 2D**). The high titers of serum anti-TB antibody indicate the promoted humoral immunity. Meanwhile, the production of CD4^+^ and CD8^+^ cells is distinctly increased in virus-mimetic co-assemblies-injected mice (**Figures 2E, F**), suggesting the enhanced cellular immune response. The elevated productions of CD4^+^ and CD8^+^ cells also verify the enhanced immunogenicity of the virus-mimetic co-assemblies. Besides, the risk of subunit vaccine based-on virus-mimetic co-assemblies is low since these subunits cannot replicate in the vaccine recipients and cause severe side effects. To sum up, MOCs exhibit the synergy with polymers, and they together enhance the immunogenicity of the antigens (50). Co-assemble of subunit antigens and molecular adjuvants provides multiple conjugation sites, increases the presentation of antigens to targeted cells, and impacts the magnitude and durability of the immunogenicity induced by antigens.

## Data Availability Statement

The original contributions presented in the study are included in the article/supplementary material. Further inquiries can be directed to the corresponding authors.

## Ethics Statement

The animal study was reviewed and approved by Institutional Animal Care and Use Committee at Peking University.

## Author Contributions

XL and KC participated in the preparation of the original draft. SL and PY participated in revision and editing. All authors listed have made a substantial, direct, and intellectual contribution to the work and approved it for publication.

## Funding

The work is supported financially by the National Natural Science Foundation of China (22101086, 21961142018, U1832220, and 51873067) and the Natural Science Foundation of Guangdong Province (2021A1515010271 and 2021A1515012024).

## Conflict of Interest

The authors declare that the research was conducted in the absence of any commercial or financial relationships that could be construed as a potential conflict of interest.

## Publisher’s Note

All claims expressed in this article are solely those of the authors and do not necessarily represent those of their affiliated organizations, or those of the publisher, the editors and the reviewers. Any product that may be evaluated in this article, or claim that may be made by its manufacturer, is not guaranteed or endorsed by the publisher.
